# Application of area scaling analysis to identify natural killer cell and monocyte involvement in the GranToxiLux antibody dependent cell‐mediated cytotoxicity assay

**DOI:** 10.1002/cyto.a.23348

**Published:** 2018-03-02

**Authors:** Justin Pollara, Chiara Orlandi, Charles Beck, R. Whitney Edwards, Yi Hu, Shuying Liu, Shixia Wang, Richard A. Koup, Thomas N. Denny, Shan Lu, Georgia D. Tomaras, Anthony DeVico, George K. Lewis, Guido Ferrari

**Affiliations:** ^1^ Department of Surgery Duke University School of Medicine Durham North Carolina; ^2^ Institute of Human Virology University of Maryland School of Medicine Baltimore Maryland; ^3^ Department of Medicine University of Massachusetts Medical School Worcester Massachusetts; ^4^ Vaccine Research Center National Institute of Allergy and Infectious Diseases, National Institutes of Health Bethesda Maryland; ^5^ Duke Human Vaccine Institute, Duke University School of Medicine Durham North Carolina

**Keywords:** natural killer cells, monocytes, antibody‐dependent cell‐mediated cytotoxicity, GranToxiLux assay, peripheral blood mononuclear cells, HIV

## Abstract

Several different assay methodologies have been described for the evaluation of HIV or SIV‐specific antibody‐dependent cell‐mediated cytotoxicity (ADCC). Commonly used assays measure ADCC by evaluating effector cell functions, or by detecting elimination of target cells. Signaling through Fc receptors, cellular activation, cytotoxic granule exocytosis, or accumulation of cytolytic and immune signaling factors have been used to evaluate ADCC at the level of the effector cells. Alternatively, assays that measure killing or loss of target cells provide a direct assessment of the specific killing activity of antibodies capable of ADCC. Thus, each of these two distinct types of assays provides information on only one of the critical components of an ADCC event; either the effector cells involved, or the resulting effect on the target cell. We have developed a simple modification of our previously described high‐throughput ADCC GranToxiLux (GTL) assay that uses area scaling analysis (ASA) to facilitate simultaneous quantification of ADCC activity at the target cell level, and assessment of the contribution of natural killer cells and monocytes to the total observed ADCC activity when whole human peripheral blood mononuclear cells are used as a source of effector cells. The modified analysis method requires no additional reagents and can, therefore, be easily included in prospective studies. Moreover, ASA can also often be applied to pre‐existing ADCC‐GTL datasets. Thus, incorporation of ASA to the ADCC‐GTL assay provides an ancillary assessment of the ability of natural and vaccine‐induced antibodies to recruit natural killer cells as well as monocytes against HIV or SIV; or to any other field of research for which this assay is applied. © 2018 The Authors. Cytometry Part A published by Wiley Periodicals, Inc. on behalf of ISAC.

There has been a recent increase of interest in evaluating HIV/SIV‐specific non‐neutralizing antibody responses, including those that mediate antibody‐dependent cell‐mediated cytotoxicity (ADCC). Focus on the non‐neutralizing Fc‐mediated antiviral activities of antibodies has largely been a result of the identification of ADCC responses as associated with the reduced risk of HIV‐1 infection observed in the RV144 clinical trial 1, reduced vertical transmission of HIV‐1 via breast milk 2, and in protection from SIV and SHIV infection in passive and active immunization trials conducted in nonhuman primates (NHPs) 3, 4, 5, 6. Several different types of nonradioisotope‐based assays have been developed to either semi‐quantitatively or quantitatively measure the HIV/SIV‐specific ADCC activity of serum, plasma, mucosal samples, or purified antibodies [reviewed in Ref. 7]. We have previously described a high‐throughput quantitative flow cytometry‐based assay that allows for detection of the proteolytic activity of Granzyme B (GzB) at the single cell level to identify target cells that have received a cytotoxic hit as a result of antigen‐specific antibody‐Fc receptor interactions 8. We and others have broadly applied this assay, the ADCC‐GranToxiLux assay (ADCC‐GTL), to the measure of HIV‐ or SIV‐specific antibody responses generated by natural infection or by active and passive immunization strategies 6, 9, 10, 11, 12, 13, 14, 15, 16, 17, 18, 19, 20, 21, 22, 23, 24, 25, 26, 27, 28, 29, 30, 31, 32, 33, 34, 35, 36, 37.

Here, we describe a simple modification of our original ADCC‐GTL assay that uses Area Scaling Analysis (ASA) 38, 39, 40 to discriminate between ADCC mediated by natural killer (NK) cells, and non‐ADCC interactions between monocytes and target cells when whole human peripheral blood mononuclear cells (PBMC) are used as a source of effector cells. Thus, this GTL‐ASA assay can be used to evaluate the ADCC activity of antibody samples while simultaneously providing ancillary assessment of the type of cells recruited by ADCC antibodies, which can be used as an additional metric to characterize the natural and vaccine‐induced ADCC responses to HIV or SIV. The incorporation of ASA to the ADCC‐GTL assay can be easily included in prospective studies, and can also often be applied to pre‐existing ADCC‐GTL datasets.

## Methods

### Human Ab Samples

HIV‐1 seronegative and seropositive plasmas were obtained from patients enrolled in studies conducted by the Duke Center for HIV and AIDS Vaccine Immunology. Vaccinee plasma samples were obtained from participants in Groups A (*n* = 2) and B (*n* = 2) of the DP6‐001 HIV vaccine phase 1 trial, a multiclade DNA prime and HIV‐envelope gp120 protein boost vaccine regimen that elicited cross‐subtype ADCC responses 10, 41. All human samples were collected in accordance with protocols approved by the local Institutional Review Boards (IRB). The HIV IgG immunoglobulin preparation (HIVIG) 42 was obtained from the NIH AIDS Research and Reagent Program. The HIV‐1 envelope specific monoclonal antibodies (mAbs) used include: C11 and A32, which bind distinct conformational epitopes in the Cluster A region of gp120 43, 44; VRC01, which binds to the CD4 binding site 45; 2G12, which binds a gp120 outer domain glycan region 46, and N10‐U1 and 7B2, which target the immunodominant region of gp41 47, 48. All mAbs were produced using recombinant techniques, and were generated using both a natural human IgG1 constant region, and IgG1 containing alanine substitutions (S298A, E333A, K334A) designed to enhance binding to Fcγ‐receptor IIIa (FcγR3A) 49. The S298A mutation also acts to decrease affinity for Fcγ‐receptor IIa and IIb (FcγR2A and B) 49. HIV‐1 negative polyclonal human IgG (Gammagard S/D; Baxter Healthcare; Westlake Village, CA) and the humanized respiratory syncytial virus (RSV) specific mAb Synagis^®^ (IgG1_k_; palivizumab; MedImmune, LLC; Gaithersburg, MD) were used as negative controls. Plasma samples were tested after fivefold serial dilutions starting at 1:50, and mAbs were tested after fivefold serial dilution resulting in a concentration range of 10 μg/ml to 3 ng/ml.

### Target Cells

A clonal isolate of the CEM.NKR_CCR5_ CD4^+^ T cell line [NIH AIDS Reagent Program, Division of AIDS, NIAID, NIH: from Dr. Alexandra Trkola 50] was used as the source of target cells. The CEM.NKR_CCR5_ cells were used after coating with a recombinant HIV‐1 gp120 protein representing the envelope of the subtype B BaL or SF162 (GenBank No. M68893 and AAT67508, respectively; Immune Technology Corp, NY), or after infection with an infectious molecular clone (IMC) virus representing HIV‐1 BaL and encoding a *Renilla* luciferase reporter gene 51. The optimal amount of gp120 for coating the target cells was determined by competing the binding of FITC‐conjugated CD4 Leu3A antibody (clone SK3; Catalog no. 340133; Final dilution 1:5, BD Bioscience, San Jose, CA) to the CD4 receptor expressed on the surface of the cell line as previously described 8. Infections with the HIV‐1 BaL IMC were performed by incubation with DEAE‐Dextran as previously described 8, and were monitored by measuring luciferase activity and determining the frequency of cells expressing intracellular p24 using standard intracellular staining methods. >75% of the viable target cells used in assays were p24 positive.

### Effector Cell Populations

PBMC obtained from a HIV‐seronegative donor with the heterozygous 158F/V and 131H/R genotypes for FcγR3A and FcγR2A, respectively, were used for all experiments except those designed to investigate how different FcγR3A and FcγR2A genotypes affect ASA. For these studies, PBMC were obtained from six HIV‐seronegative donors with the following combinations of FcγR3A and FcγR2A alleles: 158V/V 131H/H, 158F/F 131H/H, 158V/V 131R/R, 158F/F 131R/R, 158V/V 131H/R, 158F/F 131H/R. All blood donations were collected under informed consent according to the appropriate IRB‐approved protocols. Blood was processed and used or cryopreserved within 8 h of collection. Cells were counted for viability and adjusted to the proper concentration to obtain an effector to target cell ratio of 30:1. For assays performed with cryopreserved PBMC the cells were thawed and rested overnight at 2 × 10^6^ cell/ml in RPMI1640 medium supplemented with 10% FBS at 37°C and 5% CO_2_ prior to use in the assay.

For depletion experiments, NK cells or monocytes were removed from PBMC using magnetic beads coated with anti‐human CD56 antibodies or anti‐human CD14 antibodies, respectively, according to manufacturer recommended protocols (Miltenyi Biotec, Bergisch Gladbach, Germany). PBMC incubated with biotin‐coated magnetic beads (Miltenyi Biotec) were used as a negative control to account for any nonspecific depletion of cells associated with the magnetic bead isolation procedure. The purity of each depleted cell population was confirmed by flow cytometry after cell‐surface staining with aqua fluorescent LIVE/DEAD Fixable Stain (Thermo Fisher Scientific, Waltham, MA) and the following panel of antibodies: PE‐TR‐conjugated anti‐CD3 (clone S4.1/7D6; Catalog no. MHCD0317; Final dilution 1:20, Thermo Fisher Scientific, Waltham, MA), PE‐TR‐conjugated anti‐CD19 (clone SJ25‐C1; Catalog no. MHCD1917; Final dilution 1:20, eBioscience, Waltham, MA), APC‐conjugated anti‐CD32 (clone 6C4; Catalog no. 17–0329‐42; Final dilution 1:20, eBioscience/Thermo Fisher Scientific, Waltham, MA), APC‐Cy7‐conjugated anti‐CD14 (clone MφP9; Catalog no. 557831; Final dilution 1:80, BD Bioscience, San Jose, CA), PacBlue‐conjugated anti‐CD16 (clone 3G8; Catalog no. 558122; Final dilution 1:80, BD Bioscience, San Jose, CA), PE‐Cy7‐conjugated anti‐CD56 (clone NCAM16.2; Catalog no. 335809; Final dilution 1:80, BD Bioscience, San Jose, CA), FITC‐conjugated anti‐CD64 (clone 10.1; Catalog no. 555527; Final dilution 1:5, BD Bioscience, San Jose, CA), and PE‐conjugated anti‐CD89 (clone A59; Catalog no. 555686; Final dilution 1:10, BD Bioscience). Purity staining confirmed a >90% reduction in cell populations targeted for depletion. Where indicated, experiments were performed using the NK‐92 natural killer cell line, engineered to express human CD16 (CD16.NK‐92; Conkwest Inc., Encinitas, CA) or the THP‐1 monocyte like cell line (ATCC TIB‐202 cells, ATCC, Manassas, VA) as effector cells.

### ADCC‐GTL Assay

The ADCC‐GTL assay was performed as previously described 8. Briefly, coated or infected target cells were labeled with TFL4 and the viability marker NFL1 (both from OncoImmunin, Gaithersburg, MD, each at final dilution of 1:1,000). After counting and washing, 10^4^ target cells per well were added to 96‐well V‐bottom plates and incubated with the Granzyme B (GzB) substrate (OncoImmunin) and effector cells for 5 min at room temperature. The antibodies (plasma or mAbs) were then added and the plate was incubated an additional 15 min at room temperature, then for 1 h at 37°C 5% CO_2_ following centrifugation for 1 min at 300 *g*. For experiments that included staining for CD14 as described in the text, after incubation the plates were washed with 1% FBS PBS wash buffer, and PE‐conjugated anti‐CD14 antibody (clone MφP9, Catalog no. 562691; Final dilution 1:20, BD Biosciences, San Jose, CA) was added to each well. The plates were then incubated for 30 min at 4°C and subsequently washed three times with 1% FBS PBS wash buffer. Well contents were then re‐suspended in 150 µl wash buffer and acquired directly with BD Fortessa flow cytometer (BD Biosciences, San Jose, CA) within 4 h using the High Throughput Sampler (HTS, BD Biosciences). The cytometer is rigorously maintained under quality control procedures regularly performed as described by Perfetto et al. 52. The signal for each fluorophore was detected using: 1) 640 nm/40 mW laser and 660/20 filter for TFL4; 2) 405 nm/50 mW laser and 450/50 filter for NFL1; 3) 488 nm/50 mW laser and the combination of 505LP with 530/30 filters for the GzB substrate. Prior to acquisition with FACSDiva software (BD Biosciences), the area scaling factor was adjusted to ensure that cell singlets (CEM.NKR_CCR5_ CD4^+^ T cells) indicated equivalent ratios of FSC‐H and FSC‐A. Flow cytometry data analysis was performed using FlowJo 9.9.4 software (FlowJo, LLC., Ashland OR).

### ADCC‐Luc Assay

ADCC activity was also determined by a luciferase (Luc)‐based assay as previously described 17. CEM.NKR_CCR5_ target cells infected with HIV‐1 IMC encoding *Renill*a luciferase were incubated with PBMC effector cells and antibodies in ½ area opaque flat bottom plates for 30 min at room temperature in duplicate wells. The plates were then centrifuged for 1 min at 300*g*, and subsequently incubated for an additional 5.5 h at 37°C 5% CO_2_. ADCC activity, reported as percent specific killing, was calculated from the change in Relative Light Units (RLU; ViviRen luciferase assay; Promega, Madison, WI) resulting from the loss of intact target cells in wells containing effector cells, target cells, and plasma or mAb samples compared to amounts in control wells containing target cells and effector cells alone according to the following formula: percent specific killing = [(number of RLU of target and effector well—number of RLU of test well)/number of RLU of target and effector well] ×100.

### GzB^+^ Target Cell Sorting and Fluorescent Confocal Microscopy

The ADCC‐GTL assay with subsequent surface staining using PE‐conjugated anti‐CD14 was performed as described above. Well contents were transferred to flow cytometry tubes and processed using a BD FACSAria II cell sorter. Live, GzB^+^ target cells were sorted (TFL4^+^, NFL1^−^, GzB^+^) using a 100 μm nozzle, to permit collection of both single cells and stable cell–cell conjugates. The sorted cell population was concentrated by centrifugation, transferred to 8‐well chamber slides and incubated for 30 min at 37°C 5% CO_2_ to allow the cells to form a monolayer on the glass bottom of the slides. The sorted cells were then visualized using a Zeiss Laser Scanning Microscope (LSM) 5 DUO. Four laser beams with wavelengths at 405 nm, 488 nm, 543 nm, and 633 nm were used to excite the fluorophore. Representative well images were captured and analyzed using Zeiss, ZEN software (Zeiss, Oberkochen, Germany).

## Results

### Evaluation of CD14 on the Surface of ADCC Target Cells in the ADCC‐GTL Assay

**Figure 1:**
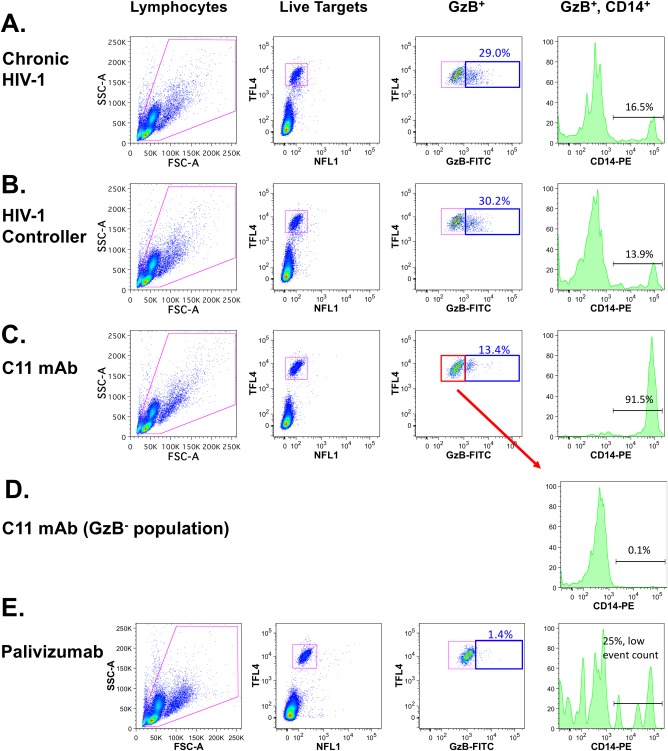
Presence of a CD14^+^ subpopulation of HIV‐1 gp120‐coated CEM.NKRCCR5 target cells in the ADCC‐GTL assay. (A) Gating strategy used for analysis of ADCC activity and evaluation of CD14 on the surface of SF162 gp120‐coated cells targeted for ADCC in ADCC‐GTL assays conducted with plasma from a chronically infected HIV‐seropositive donor, (B) plasma from an HIV‐1 virus controller, and (C) the HIV‐1 gp120 C1 region‐specific mAb C11. Histograms in the far right panels indicate the detection of CD14 on the surface of GzB^+^ target cells. (D) No CD14 was observed on the surface of GzB^−^ target cells. (E) The RSV‐specific mAb Palivizumab was used as a negative control; as expected the frequency of GzB^+^ events was low and very few CD14^+^ cells were identified.

We have previously demonstrated that the ADCC antibody responses detectable in the plasma of HIV infected individuals measured using the ADCC‐GTL assay were primarily dependent on the recruitment of NK cells present in the PBMC samples used as a source of effector cells in vitro 8. However, recent evidence for the role of monocytes as effectors in the rapid and fluorometric ADCC assay (RFADCC) 53 prompted us to reevaluate the potential contribution of monocytes to ADCC activity as measured in the GTL assay. To determine if monocytes are involved in antibody‐dependent interactions with target cells in the ADCC‐GTL assay, we modified our protocol to include surface staining of CD14 using a PE conjugated anti‐CD14 antibody after incubation of target cells with effector cells in presence of antibodies. This approach allowed us to identify a population of cells that may represent either target cells that have interacted with monocytes resulting in transfer of cell‐surface CD14 via antibody‐mediated trogocytosis as has been described for the outcome of RFADCC 53, 54, or monocytes that have obtained fluorescently labeled target cell membranes via phagocytosis. As shown in Figure 1A,B, approximately 15% of CD4^+^ T cells targeted for ADCC (cells expressing active Granzyme B, GzB^+^) were positive for cell surface CD14 after incubation with PBMC and chronic HIV seropositive plasma or plasma from a HIV‐1 controller (16.5% and 13.9% CD14^+^, respectively). In contrast, over 90% of active GzB^+^ T cell targets were identified as positive for cell‐surface CD14 when ADCC assays were performed with the HIV envelope mAb C11, specific for epitope within the 7‐stranded‐ß sandwich of gp120 Cluster A epitope region 40, 55 (Fig. 1C). Importantly, the CD14^+^ cell population was restricted to those target cells positive for active GzB, no CD14 was observed on target cells that were GzB^‐^ (Fig. 1D) or in wells containing target cells only (not shown). Low background levels of ADCC activity, and thus very few CD14^+^ target cells (<50 total CD14^+^ GzB^+^ cells) were observed with the RSV‐specific mAb Palivizumab (Fig. 1E). These data demonstrate that specific antibody‐dependent interactions between monocytes and target cells can contribute to the total events measured as ADCC activity in the ADCC‐GTL assay.

### Conventional NK‐Dependent ADCC and Monocyte‐Dependent Non‐ADCC Activity Can Be Differentiated by Unique Area Scaling Properties

**Figure 2:**
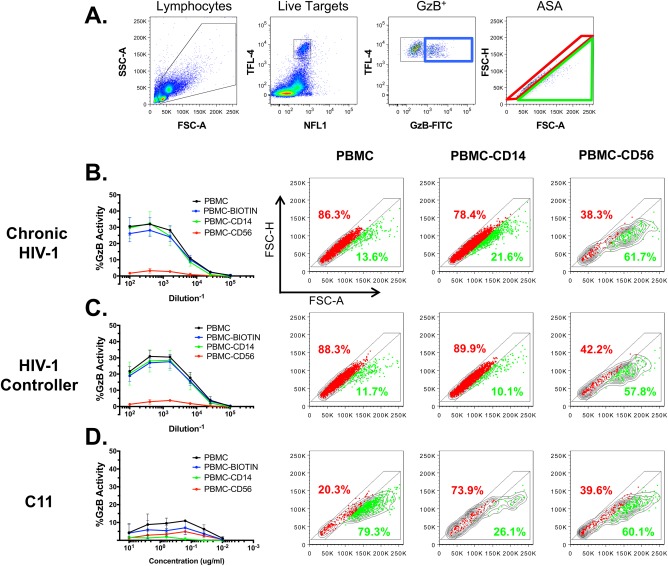
Application of Area Scaling Analysis (ASA) to the ADCC‐GTL assay. (A) Gating strategy used for ASA of GzB^+^ SF162 gp120‐coated target cells in the modified ADCC‐GTL assay. This gating strategy was used to identify singlet (red gate) and non‐singlet (green gate) GzB^+^ target cell events in ADCC‐GTL assays performed with whole PBMC, PBMC depleted of CD14^+^ cells, PBMC depleted of CD56^+^ cells, and control PBMC depleted with biotin as sources of effector cells. ADCC antibody samples were plasma collected from an HIV‐1 infected donor during chronic infection (B), plasma from an HIV‐1 virus controller (C), and the C11 mAb (D). Data in the line graphs represents mean and SD from 3 independent experiments, and flow cytometry dot plots (B–D) include concatenated data from all 3 experiments.

Monocytes and cell conjugates can be discriminated from single target cells by unique forward light scatter properties. We applied ASA to GzB^+^ events in the ADCC‐GTL assay to explore whether monocytes or monocyte–target cell conjugates were being included in the GzB^+^ gate used to define ADCC activity. We defined two gates using ASA (Fig. 2A). The first gate (shown in red) includes cells with equivalent ratios of forward scatter height (FSC‐H) and area (FSC‐A). Events that fall within this gate are typically considered cell singlets 38, 39, 40. The second gate (show in green) includes events that are biased toward higher FSC‐A relative to FSC‐H, a property associated with amorphously shaped cells or cell conjugates 38, 39, 40. We applied this new gating strategy to ADCC assays performed with whole PBMC, PBMC depleted of CD14^+^ cells (removing monocytes), and PBMC depleted of CD56^+^ cells (removing NK cells). Depletion with biotin‐coated beads was used as a negative control to account for any nonspecific depletion of cells associated with the magnetic bead isolation procedure. Depletion of CD56^+^ cells largely abrogated ADCC activity (>85% reduction of maximum observed activity) of plasma collected from an HIV‐1 infected patient during the chronic stage of disease (Fig. 2B, left panel) and from an HIV‐1 controller (Fig. 2C, left panel), indicating a predominant dependence on NK cells for ADCC by these plasma samples. We next applied ASA to the GzB^+^ target events for each of these assay conditions (Fig. 2B,C, right panels) focusing on the dilution of plasma that indicated the maximum observed ADCC response according to previous titration experiments. For assays conducted with whole PBMC we observed frequencies of cells in the singlet gate and nonsinglet gate (monocyte/cell conjugates) that were consistent with the contribution of NK cells and monocytes to total ADCC activity as determined by depletion of these cell subsets (>85% singlets, <15% nonsinglets). Similar distributions of singlet and nonsinglet events were observed when the assays were performed with PBMC depleted of CD14^+^ cells. In contrast, when the assays were conducted with PBMC depleted of CD56^+^ cells the ASA indicated a bias toward events in the nonsinglet gate. These data suggest that the GzB^+^ events located in the singlet gate represent cells targeted by NK cells, and events in the nonsinglet gate represent cells targeted by monocytes. In support of this, depletion experiments indicated that GzB activity measured by ADCC‐GTL in assays performed using the C11 mAb required the presence of monocytes (Fig. 2D, left panel) and the ASA analysis of assays conducted with whole PBMC indicated a bias toward events in the nonsinglet gate, whereas when the assay was performed after depletion of CD14^+^ monocytes we observed a switch to the majority of the GzB^+^ events falling into the singlet gate (Fig. 2D, right panels). This indicates that the C11 mAb used for these experiments preferentially recruited monocytes when they were present in the effector cell population, whereas with depletion of monocytes, NK cells were recruited.

We further explore the ability of plasma antibodies to recruit a CD16^+^ NK cell line (CD16.NK‐92 cells) or the THP‐1 monocyte cell line as effector cells by applying ASA to ADCC‐GTL assays performed using these two types of effector cells. We found that over 90% of the GzB^+^ events fall within the gate defined as singlets when CD16.NK‐92 cells are used as effectors, while nearly 90% of the GzB^+^ events fall with the nonsinglet gate when THP‐1 monocytes were used as effector cells (Supporting Information Fig. S1).

Collectively, these data suggest that ASA applied to the GzB^+^ events collected with the ADCC‐GTL assay can be used to indicate the relative contribution of NK cells (singlet events) and monocytes (nonsinglet events) to the total activity measured using the ADCC‐GTL assay.

### Monocytes Can Form Ab‐Dependent Cell‐to‐Cell Interactions with gp120‐Coated Target Cells in the ADCC‐GTL Assay

**Figure 3:**
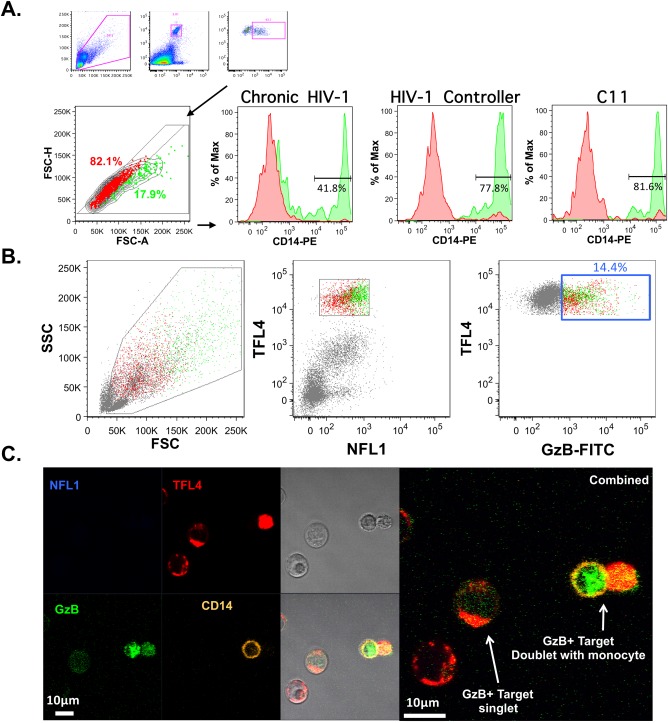
Monocytes can form Ab‐dependent cell‐to‐cell interactions with gp120‐coated target cells in the ADCC‐GTL assay. GzB^+^ target cells that fall within the singlet gate do not have cell‐surface CD14, while a large portion of GzB^+^ cells within the non‐singlet gate have cell‐surface CD14. (A) Gating strategy and histograms indicating detection of CD14 in the singlet gate (red histograms) and the non‐singlet gate (green histograms) for ADCC‐GTL assays performed with HIV‐seropositive plasma and the C11 mAb as indicated. (B) An ADCC‐GTL assay was performed with PBMC effector cells, gp120‐coated target cells, and the C11 mAb. The GzB^+^ events were sorted using the gates shown (B), and evaluated by confocal microscopy after staining for CD14 (C). Both GzB^+^ singlet events and monocyte‐target cell conjugates were observed.

Our effector cell depletion experiments suggested that GzB^+^ target cells that have undergone Ab‐dependent interactions with monocytes have unique area scaling properties when compared to GzB^+^ target cells that have interacted with NK cells. To further probe these monocyte and target cell interactions, we combined surface staining for CD14, after completion of the ADCC assay incubation, with ASA to determine if the CD14 positive subpopulation we have identified is restricted to the nonsinglet gate. Using the plasma samples collected from HIV‐1 infected individuals and the C11 mAb as the sources of ADCC‐mediating antibodies we found that the majority (>70%) of GzB^+^ target cells within the nonsinglet gate were CD14^+^ (Fig. 3A, green events and histograms), whereas the GzB^+^ target cells that fell within the singlet gate did not express cell‐surface CD14 with any source of Ab (Fig. 3A, red events and histograms). The presence of CD14 on the surface of GzB^+^ can occur via trogocytosis or phagocytosis, but may also represent the formation of stable cell–cell conjugates of target cells and monocytes. To determine if Ab‐dependent cell–cell conjugates were an outcome of interactions between monocytes and target cells we performed the ADCC‐GTL assay with the C11 mAb, modified to include CD14 surface staining after the 1 h incubation, and sorted GzB^+^ target cells according to the gating strategy shown in Figure 3B. Confocal microscopy was then performed on the sorted GzB^+^ target cells as shown in Figure 3C. The combined field shows that monocytes (CD14^+^) and live target cells (NFL1^−^ TFL4^+^) can form stable cell–cell conjugates. We also confirmed that monocytes are able to acquire TFL4 from target cells during these cell–cell conjugate interactions, likely via the process of trogocytosis. Confocal microcopy of assays performed with the C11 mAb indicated the transfer of TFL4 from the gp120‐coated target cells to CD14^+^ monocytes (Supporting Information Fig. S2). This transfer was observed when either whole PBMC (Supporting Information Fig. S2 panel A) or purified human monocytes (Supporting Information Fig. S2 panel B) were used as effectors, suggesting that ADCC‐mediated NK‐dependent apoptosis is not a prerequisite for monocytes to acquire TFL4 from target cells. In addition, nonconjugated monocytes positive for TFL4 were also observed, suggesting the TFL4 fluorescent dye is retained by monocytes after separation of monocyte–target cell conjugates.

### NK Cell ADCC‐GTL Activity as Measured by Area Scaling Analysis Correlates with Killing of HIV‐1 Infected Cells in the Luciferase‐Based (ADCC‐Luc) Assay

**Figure 4:**
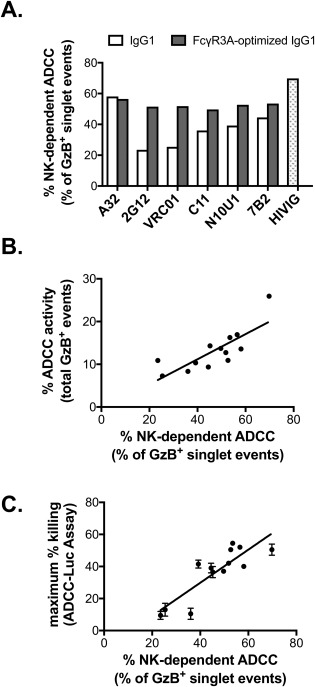
NK cell‐mediated ADCC is associated with specific lysis of HIV‐infected cells. (A) Optimization of antibody Fc regions for binding to FcγR3A results in increased % NK cell dependent ADCC directed against HIV‐infected cells as measured by ASA in the modified ADCC‐GTL assay. (B) Total ADCC activity of the antibody panel shown in panel A measured using the ADCC‐GTL assay is positively associated with % NK cell dependent ADCC (singlet gate). (C) The % NK cell dependent ADCC against HIV‐infected targets in the ADCC‐GTL assay correlates with the maximum specific killing of HIV‐infected target cells as measured by the ADCC‐Luc assay. ADCC‐Luc assays were performed in duplicate and are reported as mean and standard deviation.

Having demonstrated that both conventional NK‐dependent ADCC, and non‐ADCC activity resulting from monocyte–target cell interactions including conjugate formation and trogocytosis, contribute to the total activity measured by the flow cytometry‐based ADCC‐GTL assay we next sought to evaluate the association of NK‐mediated and monocyte‐mediated activity as determined by ASA with lysis of HIV‐1 infected cells in the ADCC‐Luc assay. We used a panel of six HIV‐specific monoclonal human IgG antibodies produced via recombinant techniques as human IgG1, and IgG1 with an engineered Fc region that includes alanine substitutions to enhance binding to FcγR3A (S298A, E333A, K334A), as sources of ADCC‐mediating mAbs 49. We also used the polyclonal HIV‐specific IgG preparation, HIVIG. The RSV‐specific human IgG1 mAb Palivizumab and Gammagard^®^ polyclonal human IgG preparation were used as negative controls. As expected, only low background levels of ADCC activity was observed with our negative controls (Palivizumab, 1% GzB activity in ADCC‐GTL, 0% specific killing in ADCC‐Luc; Gammagard, 0.7% GzB activity in ADCC‐GTL, 1.5% specific killing in ADCC‐Luc). HIV‐1_BaL_‐infected CD4^+^ T cells were used as target cells for both the ADCC‐GTL and ADCC‐Luc assays, and PBMC from a HIV‐seronegative donor with the heterozygous FcγR3A 158F/V phenotype served as the source of effector cells 55. ASA was applied to ADCC‐GTL data that represented the highest observed total ADCC activity within the dilutions curves tested for each antibody. As expected, optimization of the antibody Fc region to improve binding to FcγR3A resulted in higher levels of NK‐cell mediated ADCC (percentage of live, GzB^+^ singlet events) as indicated by GTL‐ASA for five out of six tested mAbs (Fig. 4A). We observed a positive correlation between the ability of antibodies to recruit NK cells for ADCC activity with total ADCC activity in the ADCC‐GTL assay (Fig. 4B, Spearman *r* = 0.81, *P* < 0.001), as well as with maximum observed specific killing of HIV‐infected target cells using the ADCC‐Luc Assay (Fig. 4C, Spearman *r* = 0.80. *P* < 0.001) 17. In contrast, we observed a negative correlation between the ability of antibodies to recruit monocytes and the maximum observed specific killing of HIV‐infected target cells in the ADCC‐Luc Assay (Supporting Information Fig. S3, Spearman *r*=‐0.80, *P* < 0.001), suggesting that recruitment of monocytes may result in trogocytosis, phagocytosis, slower killing of target cells, or other antiviral responses that are not discernable in the ADCC‐Luc Assay. These results demonstrate that the GTL‐ASA assay data can be used to inform on the type of effector cells recruited by antibodies, thus providing an additional metric for evaluation of HIV or SIV‐specific Fc‐mediated antibody responses.

### ASA Is Unable to Differentiate Donor Cells Based on FcγR3A and FcγR2A Single Nucleotide Polymorphisms

**Figure 5:**
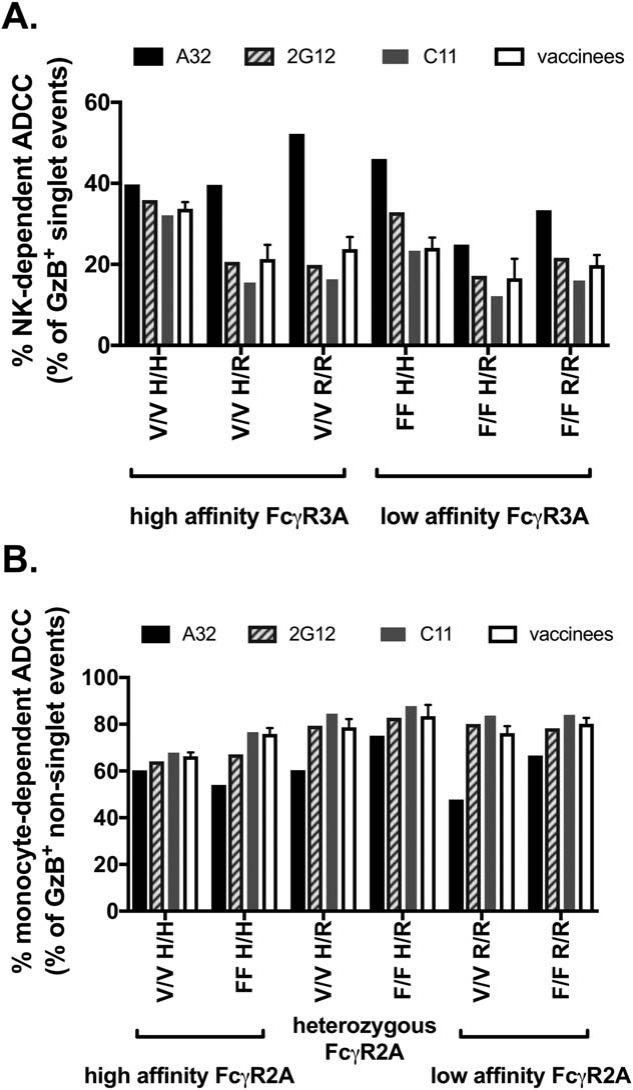
ASA is unable to differentiate donor cells based on FcγR3A and FcγR2A single nucleotide polymorphisms. The ADCC‐GTL assay was performed using HIV‐specific IgG mAbs, vaccinee plasma samples (*n*=4), and PBMC collected from 6 different donors, all with unique allelic combinations of common FcγR3A and FcγR2A single nucleotide polymorphisms. The percentage of the total maximum ADCC response attributed to NK cells (A) and monocytes (B) was determined by ASA. Donor FcγR3A and FcγR2A are grouped by phenotype as indicated by the brackets below the X axis in panel A and B, respectively. Data for the vaccinee samples (*n*=4) represents the mean and standard deviation.

We have demonstrated that ASA‐GTL can be used to identify differences in the ability of antibodies to recruit NK cells or monocytes for ADCC when applied within assays performed with PBMC from the same donor. We next asked whether ASA could be used to identify differential recruitment of NK cells or monocytes resulting from single nucleotide polymorphisms for FcγR3A and FcγR2A. To test this possibility, we performed ASA‐GTL assays using cryopreserved PBMC collected from six donors with the following alleles for FcγR3A and FcγR2A, respectively: 158V/V 131H/H, 158F/F 131H/H, 158V/V 131R/R, 158F/F 131R/R, 158V/V 131H/R, and 158F/F 131H/R. The percentages of total ADCC attributed to NK cells (Fig. 5A) or monocytes (Fig. 5B) at the antibody concentration or plasma dilution representing the maximum observed ADCC responses were determined by ASA of GzB^+^ target as described above. In ADCC assays performed with HIV‐specific IgG1 mAbs A32, 2G12, and C11, or with plasma samples collected from recipients of a candidate DNA‐prime protein‐boost HIV‐vaccine 10, 41, we found that NK cell‐mediated ADCC (Fig. 5A) did not rank‐order as expected based on FcγR3A genotype 56, 57, 58, 59, 60, 61. Specifically, we observed no bias toward recruitment of NK cells in assays performed with PBMC from donors with the higher affinity 158V/V allelic variant of FcγR3A when compared to donors with the lower affinity 158F/F variant. Moreover, we did not observe any discernable impact of FcγR2A alleles on monocyte‐mediated ADCC (Fig. 5B). Thus, ASA can provide information on antibodies when assays are performed with the same PBMC, but is unlikely to provide information on characteristics of donor effector cells for assays performed with a set panel of antibodies and PBMC from multiple donors representing different FcγR phenotypes. This may be related to intrinsic differences in frequencies and activation states of circulating NK cells and monocytes amongst donors.

## Discussion

Multiple assay methodologies have been described and applied to the evaluation of HIV or SIV‐specific ADCC activity [reviewed in Ref. 7]. Assays such as the newly developed FcγR3A cell line reporter assays (Promega ADCC Reporter Bioassay) and the CD107a degranulation/intracellular cytokine assay 62 measure ADCC by detecting signaling through Fc receptors, or cellular activation and/or production of antiviral and cytolytic factors by Fc receptor‐bearing effector cells. Thus, these assays are mainly intended to evaluate the effect of ADCC responses at the level of the effector cell populations. Other assays such as the ADCC‐GTL assay 8, ADCC‐Luc assay 17, 63, and the classic ^51^Cr release assay 64 assess the effect of ADCC responses on the target cell populations by detecting the initiation of apoptosis, or by measuring cytolysis. Thus, each of these assays provides information on only one of the critical components of an ADCC event; either the effector cells involved, or the resulting effect on the target cell. Obtaining information on both cell populations typically requires depletion of specific subsets of effector cells from PBMC, the use of purified effector cell populations, or the use of a combination of ADCC assay methodologies. Here we have described a simple modification of the ADCC‐GTL assay that applies ASA to assess the ability of ADCC antibodies to interact with NK cells and monocytes present in the PBMC used as a source of effector cells. The inclusion of ASA in the evaluation of the results obtained with ADCC‐GTL assay allows for simultaneous quantification of target cell recognition, and the relative recruitment of monocytes or NK cells by ADCC antibodies.

The involvement of monocytes in HIV‐specific ADCC was highlighted by Kramski et al. through a detailed characterization of the RFADCC assay 53. Interestingly, they found that the cell population typically regarded as killed target cells in this assay is primarily comprised of CD14^+^ cells from the effector cell population that have obtained some of fluorescent dye used to identify the target cells. This may happen either as a result of monocyte phagocytosis or by cell surface membrane exchange via trogocytosis. Additional work is required to fully define the biological mechanism detected with the RFADCC assay, but it is clear from the work of Kramski et al. that it does not strictly and solely measure NK cell‐mediated target cell lysis as initially thought. Antibody responses measured by the RFADCC assay have been correlated with reduced infection risk and virus control 2, 4, 65, 66, 67, 68, 69, 70, suggesting that antibody‐mediated recruitment of monocytes may be an important component of HIV‐specific immune responses warranting further study.

We have previously demonstrated that whole PBMC and purified NK cells have similar ability to mediate ADCC as measured by the ADCC‐GTL assay, and the majority of the observed HIV‐specific ADCC activity was lost when PBMC were depleted of CD56^+^ cells or CD16^+^ cells 8. These data suggested that NK cells were the predominant cell type involved in ADCC as detected by the ADCC‐GTL assay using plasma from naturally HIV‐1‐infected viremic subjects or SIV infected nonhuman primates. To more carefully evaluate the possible role of monocytes, we modified the ADCC‐GTL assay method to include cell surface staining with a fluorescent anti‐CD14 antibody as described for characterization of the “killed” target cells in the RFADCC assay 53. We found that the majority of target cells positive for active GzB after incubation with HIV‐1 seropositive plasma lacked cell‐surface CD14 as expected, whereas a population of GzB^+^ CD14^+^ cells was observed when assays were conducted with the Cluster A‐region specific mAb C11. We further confirmed these findings by applying ASA in combination with specific depletion of NK cells or monocytes from PBMC used as a source of effector cells. We found that the population of GzB^+^ target cells within the singlet gate lacked CD14 and were primarily dependent on the presence of NK cells in the effector cell population. In contrast, the population biased toward higher FSC‐A relative to FSC‐H, and associated with amorphously shaped cells or cell conjugates, was primarily dependent on the presence of monocytes. These nonsinglet events were highly enriched for CD14^+^ events within the target cell gate, and confocal microscopy demonstrated the presence of GzB^+^ target cell–CD14^+^ monocyte cell conjugates within this population. Thus, we propose that ASA can be applied to the ADCC‐GTL assay to identify ADCC mediated by NK cells (TFL4^+^ GzB^+^ singlet events) and target cells that have interacted with monocytes (TFL4^+^ GzB^+^ nonsinglet events). Our data also demonstrated that while ASA can provide information on antibody‐mediated recruitment of NK cells and monocyte when assays are performed with the same PBMC, it is unable to differentiate donor cells based on FcγR3A and FcγR2A single nucleotide polymorphisms. This suggests that ASA will be most informative when applied to datasets collected using the same source of effector cells, and not across datasets using effector cells collected from different donors.

Using a panel of HIV‐specific mAbs, we demonstrated that mAbs optimized for binding to FcγR3A yielded higher NK‐mediated ADCC activity compared to monocyte‐mediated trogocytosis or phagocytosis. We also found that NK‐mediated ADCC was positively associated with higher levels of total ADCC activity among the mAb panel, and NK‐mediated ADCC measured in the ADCC‐GTL assay was positively correlated with lysis of HIV‐infected target cells as measured by the ADCC‐Luc assay. Additional work to explore how GTL‐ASA can inform our understanding of HIV/SIV specific ADCC responses is ongoing. Importantly, we recently applied GTL‐ASA to a study aimed at identifying correlates of protection from SHIV challenge following active immunization in a NHP model 6. In this study, rhesus macaques were immunized with a poxvirus prime and protein boost vaccine regimen designed to improve on the efficacy observed for the RV144 clinical trial 1, 71. A comprehensive analysis of immune correlates of infection risk identified the maximum NK cell‐mediated ADCC activity (measured using ASA‐GTL) against target cells coated with a subtype E gp120 isolate as a component of the immune response correlated with decreased risk of infection (concordance index 0.7, *P* < 0.05) after repeated low dose challenges. These data suggest that ADCC mediated by NK cells may contribute to protection from SHIV infection in the NHP model system. We are currently studying the biology of monocyte recruitment measured by GTL‐ASA as our recent data suggests that ADCC mediated by NK cells and antibody‐dependent recruitment of monocytes have distinct physiological roles in immune responses to HIV/SIV infection. The interaction with monocytes may not lead to rapid killing of target cells, but may result in trogocytosis, phagocytosis, delayed killing, or other yet unknown immune effector functions. The inclusion of ASA in the ADCC‐GTL assay can be used to help define the unique immunologic functions of NK cell‐mediated and monocyte‐mediated antibody responses in protection from HIV/SIV infection, or for the control of viremia and disease progression. GTL‐ASA can also be used to explore the antibody‐dependent contribution of NK cells and monocytes to immune responses directed against any other disease or malignancy for which this assay platform is applied.

## Supporting information

Additional supporting information may be found in the online version of this article.

Additional MIFlowCyt

Additional FigureS1

Additional FigureS2

Additional FigureS3
