# Supplementary material for: Application of area scaling analysis to identify natural killer cell and monocyte involvement in the GranToxiLux antibody dependent cell‐mediated cytotoxicity assay
Source: Cytometry A. 2018 Mar 2;93(4):436–47. doi: 10.1002/cyto.a.23348 (PMC5969088; doi:10.1002/cyto.a.23348)
Supplement: Supplementary file 1 — Additional MIFlowCyt [file CYTO-93-436-s001.pdf]

Cytometry Part A
Author Checklist: MIFlowCyt-Compliant Items

Requirement	Please Include Requested Information
1.1. Purpose	Modification of the GranToxiLux ADCC assay to include Area Scaling Analysis (ASA) for qualitative assessment of antibody recruitment of NK cells and monocytes
1.2. Keywords	ADCC, Granzyme B, natural killer (NK cells), monocytes
1.3. Experiment variables	Experiment variables described in the materials and methods and results section of the manuscript.
1.4. Organization name and address	Most experiments performed at: Duke University School of Medicine, Durham, NC 27710.<br><br>Flow sorting experiments performed at: Institute of Human Virology, University of Maryland School of Medicine, 725 West Lombard Street, Baltimore, MD 21201
1.5. Primary contact name and email address	Justin Pollara: justin.pollara@duke.edu (Duke University School of Medicine) or, Chiara Orlandi: COrlandi@ihv.umaryland.edu (IHV)
1.6. Date or time period of experiment	08/2014 – 09/2017
1.7. Conclusions	The inclusion of area scaling analysis (ASA) in the GranToxiLux ADCC assay allows simultaneous quantification of ADCC activity at the target cell level, and assessment of the contribution of natural killer cells and monocytes to the total observed ADCC activity when whole human peripheral blood mononuclear cells are used as a source of effector cells.
1.8. Quality control measures	The Duke Cytometer has been optimized and maintained according the procedures described by Perfetto and colleagues (Perfetto SP, Ambrozak D, Nguyen R, Chattopadhyay PK, Roederer M. Quality assurance for polychromatic flow cytometry using a suite of calibration beads. Nat Protoc 2012;7:2067-79.) In addition, both the Duke Cytometer and the IHV cell sorter are evaluated daily using Cytometer Setup & Tracking Breads – BD Biosciences.
2.1.1.1. (2.1.2.1., 2.1.3.1.) Sample description	Human plasma samples and human monoclonal antibodies used for this study are described in the Materials and Methods and Figure Legends.
2.1.1.2. Biological sample source description	Human T cell line ($CEM.NKR_{CCR5}$) and human PBMC; details in Materials and Methods
2.1.1.3. Biological sample source organism description	Human origin cell lines and primary cells from blood.
2.1.2.2. Environmental sample location	-80°C storage for plasma/mAb samples, PBMC stored in LN2
2.3. Sample treatment description	Detailed descriptions are in the Material and Methods section of the manuscript
2.4. Fluorescence reagent(s) description	Fluorescent reagents are described in the Materials and Methods section of the manuscript

3.1. Instrument manufacturer	Becton Dickinson (BD)
3.2. Instrument model	LSR Fortessa (Duke University) and FACSAria II cell sorter (IHV)

3.3. Instrument configuration and settings

Duke BD Fortessa:

Cytometer Configuration	
Software Version	BD FACSDiva 8.0.1
Export Time	7/24/17 9:52
User	
Cytometer	LSRFortessa
Serial Number	H64779400025
Configuration Name	F230
Folder Name	Current Configurations
Comments	
Window Extension	10

Laser Name	Type	Wavelength	Power	Detector Array	Detector	Channel	Mirror	Filter	Parameter	Fsc Channel	Reference	Position
Red	Red	640	40	Trigon	A	14	750 LP	780/60 BP	APC-Cy7			3
					B	13	685 LP	730/45 BP	Alexa Fluor 700			
					C	12		660/20 BP	APC			
Blue	Blue	488	50	Trigon	A	3	685 LP	710/50 BP	PerCP-Cy5-5	0	Y	1
					B	2	505 LP	530/30 BP	FITC			
					C	1		488/10 BP	SSC			
Violet	Custom	405	50	Octagon	A	6	750 LP	780/60 BP	BV786			2
					B	5	735 LP	740/35 BP	BV745			
					C	4	690 LP	710/50 BP	BV711			
					D	15	630 LP	660/20 BP	BV650			
					E	16	600 LP	610/20 BP	BV605			
					F	17	550 LP	585/15 BP	BV570			
					G	18	505 LP	525/50 BP	BV510			
					H	19		450/50 BP	BV421			
561 Yellow-Green	Custom	561	50	Octagon	A	11	750 LP	780/60 BP	PE-Cy7			4
					B	10	685 LP	710/50 BP	PE-Cy5-5			
					C	9	635 LP	660/20 BP	PE-Cy5			
					D	8	600 LP	610/20 BP	PE-Texas Red			
					E	7		582/15 BP	PE			

IHV BD FACSAria II Sorter:

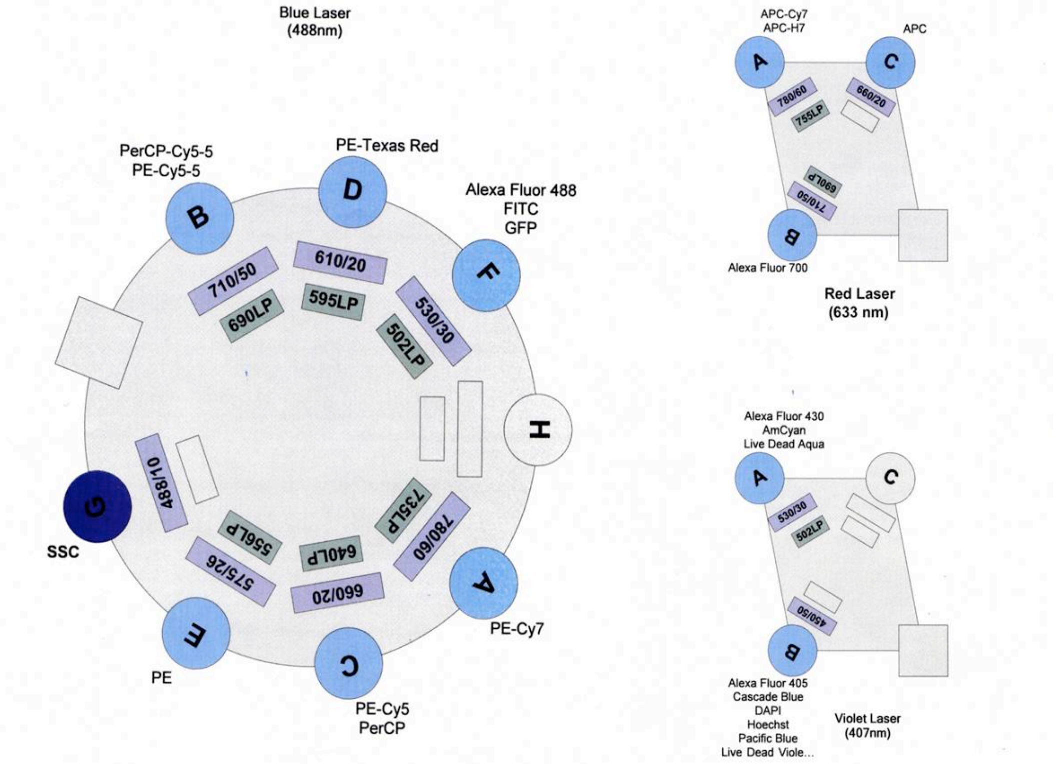

4.1. List-mode data files	The repository identifier:<br><br>http://flowrepository.org/id/FR-FCM-ZYD5
4.2. Compensation description	Anti-Mouse Ig, κ/Negative Control Compensation Particles Set (BD Biosciences cat. 552843) used for conjugated fluorescent mAbs. TFL4 compensation used TFL4-stained CEM.NKR_{CCR5} cells for positive compensation controls. NFL1 compensation used NFL1 stained permeabilized and fixed CEM.NKR_{CCR5} cells for positive compensation controls. Unstained CEM.NKRCCR5 cells were used as negative compensation controls for TFL4 and NFL1.
4.3. Data transformation details	Log transformation of fluorescence channels, lin transformation of scatter characteristics (FlowJo Software v9/9.4)
4.4.1. Gate description	Gating strategies and boundaries are indicated in Figures presented in the manuscript.
4.4.2. Gate statistics	Frequency of Parent/gate, Median Fluorescent Intensity
4.4.3. Gate boundaries	Gating strategies and boundaries are indicated in Figures presented in the manuscript.
