# Supplementary figures and images for: Application of area scaling analysis to identify natural killer cell and monocyte involvement in the GranToxiLux antibody dependent cell‐mediated cytotoxicity assay
Source: Cytometry A. 2018 Mar 2;93(4):436–47. doi: 10.1002/cyto.a.23348 (PMC5969088; doi:10.1002/cyto.a.23348)

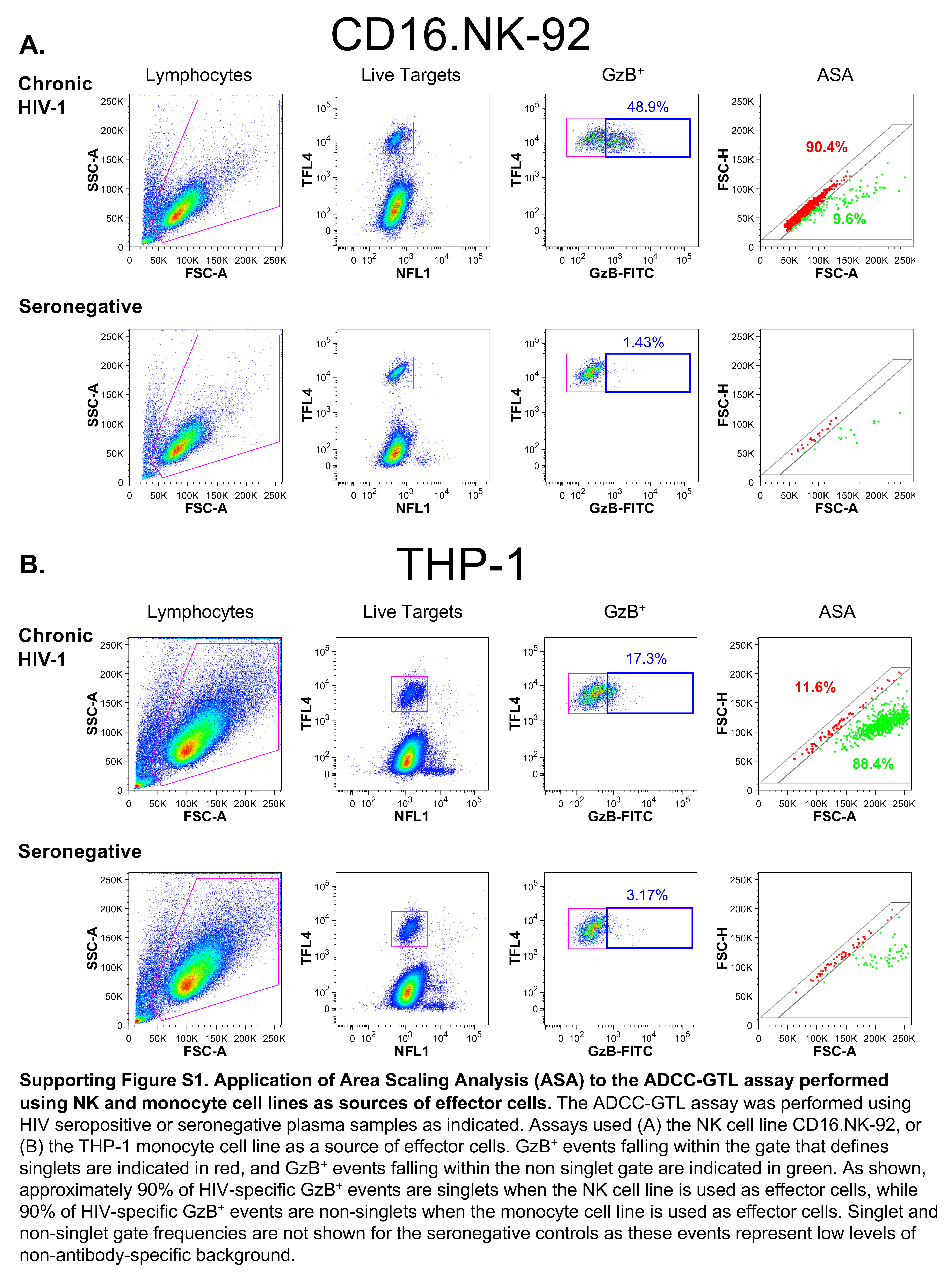

Supplement: Supplementary file 2 — Additional FigureS1 [file CYTO-93-436-s002.tif]

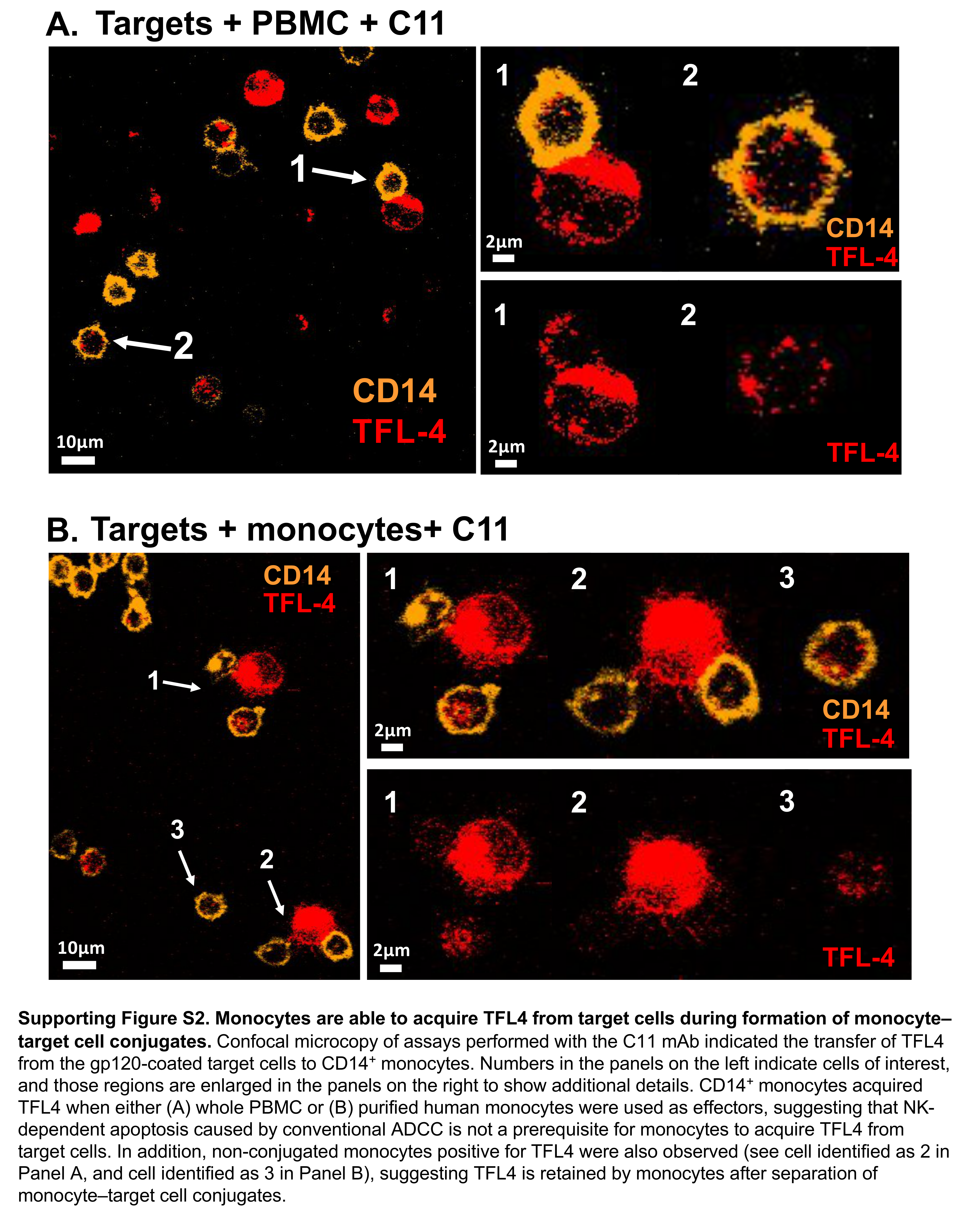

Supplement: Supplementary file 3 — Additional FigureS2 [file CYTO-93-436-s003.tif]

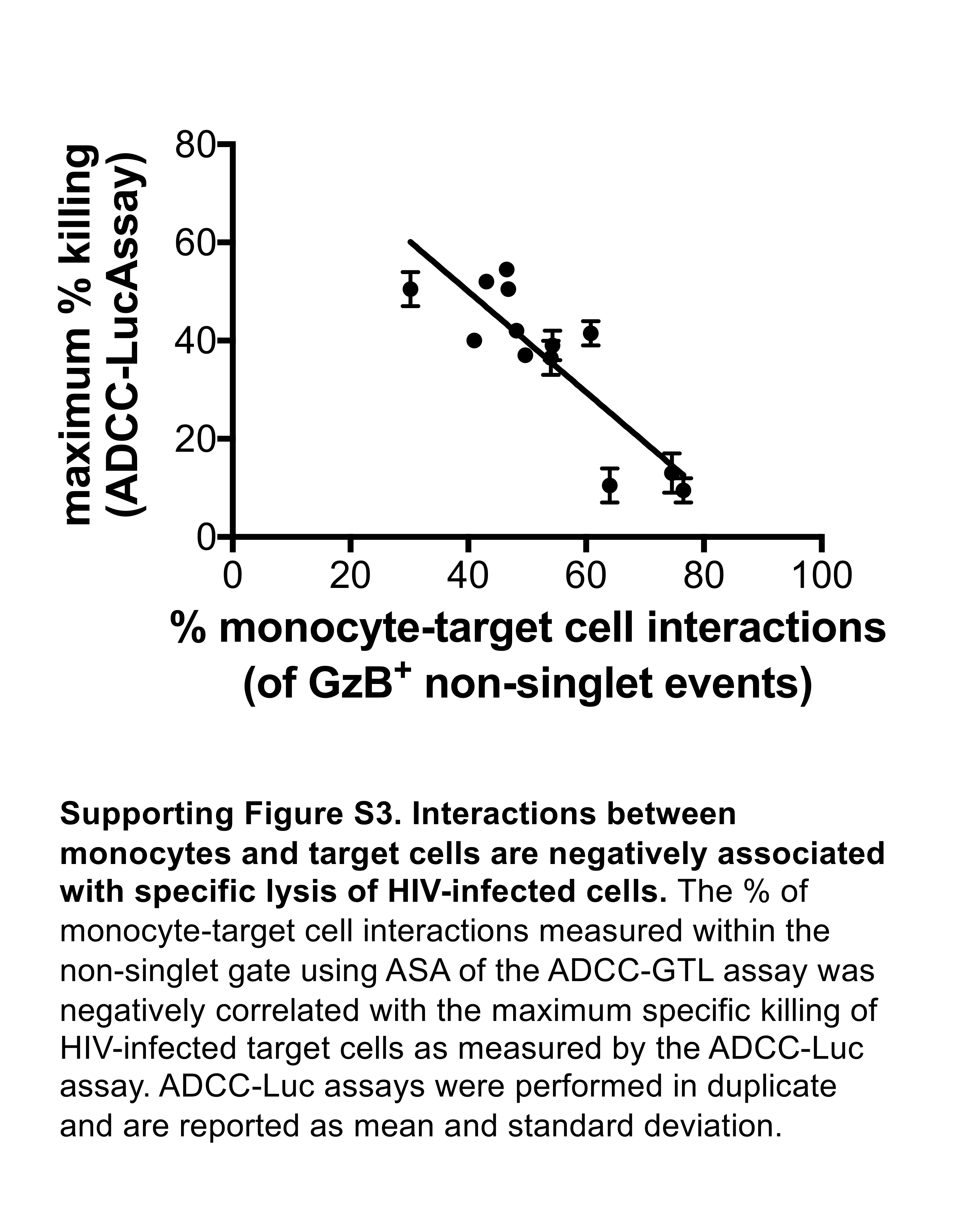

Supplement: Supplementary file 4 — Additional FigureS3 [file CYTO-93-436-s004.tif]
